# THE INTERPLAY BETWEEN GENETIC VARIANTS IN MIR146A AND ABCG2 GENES IS ASSOCIATED WITH HUMAN SURVIVAL

**DOI:** 10.1093/geroni/igae098.3471

**Published:** 2024-12-31

**Authors:** Anatoliy Yashin, Deqing Wu, Konstantin Arbeev, Hongzhe Duan, Rachel Holmes, Igor Akushevich, Svetlana Ukraintseva

**Affiliations:** Duke University, Durham, North Carolina, United States; Duke University, Durham, North Carolina, United States; Duke University, Durham, North Carolina, United States; Duke University, Durham, North Carolina, United States; Duke University, Durham, North Carolina, United States; Duke University, Durham, North Carolina, United States; Duke University, Durham, North Carolina, United States

## Abstract

The ABCG2 gene is well-known for its role in developing drug resistance and for its impact on the survival of cancer patients. To modulate the effects of ABCG2 on health and survival different intervention strategies were suggested. Among them, the use of non-coding RNAs that are involved in posttranscriptional modification of gene expression was suggested. MiR146a is known as a posttranscriptional regulator of expressions of many genes involved in aging, health, and survival traits. In this paper, we evaluate the role of interplay between variants in miR146a and ABCG2 genes in human survival. We apply the logistic regression model with the interaction term to the analysis of Framingham Heart Study data to test the null hypothesis that the interplay between variants in miR146a and ABCG2 genes is not associated with human survival. As an outcome we use the survival trait, Sur1, defined by dichotomizing a person’s lifespan, LS, as follows: Sur1=1, if LS >=85 years; Sur1=0 if LS < 85. We used the clumping procedure to reduce the number of tests and increase the Bonferroni correction threshold (BCT). The analysis detected a statistically significant association of interaction between rs13120400 in the ABCG2 gene and rs2910163 in the miR146 gene, β=-0.46. p=3.74E-03, BCT = 1.00E-02. Interactions of rs13120400 also showed nominally statistically significant associations with two other SNPs from the miR146a gene. rs2910164: β=-0.38, p=1.96E-02; and rs2961920: β=-0.36, p=2.45E-02. It turns out that all four SNPs are well known in scientific literature and play important roles in many health and longevity-related traits.

